# Early winners and losers in dialysis center pay-for-performance

**DOI:** 10.1186/s12913-017-2764-4

**Published:** 2017-12-08

**Authors:** Milda R. Saunders, Haena Lee, Marshall H. Chin

**Affiliations:** 1 University of Chicago Medicine, 5841 S. Maryland, MC 2007, Chicago, IL 60637 USA; 2 Institute for Social Research, University of Michigan, 426 Thompson St., #3428, Ann Arbor, MI USA

**Keywords:** End stage renal disease, Quality improvement, Pay-for-performance, Racial disparities

## Abstract

**Background:**

We examined the association of dialysis facility characteristics with payment reductions and change in clinical performance measures during the first year of the United States Centers for Medicare & Medicaid Services (CMS) End Stage Renal Disease Quality Incentive Plan (ESRD QIP) to determine its potential impact on quality and disparities in dialysis care.

**Methods:**

We linked the 2012 ESRD QIP Facility Performance File to the 2007–2011 American Community Survey by zip code and dichotomized the QIP total performance scores—derived from percent of patients with urea reduction rate > 65, hemoglobin < 10 g/dL, and hemoglobin > 12 g/dL—as ‘any’ versus ‘no’ payment reduction. We characterized associations between payment reduction and dialysis facility characteristics and neighborhood demographics, and examined changes in facility outcomes between 2007 and 2010.

**Results:**

In multivariable analysis, facilities with any payment reduction were more likely to have longer operation (OR 1.03 per year), a medium or large number of stations (OR 1.31 and OR 1.42, respectively), and a larger proportion of African Americans (OR 1.25, highest versus lowest quartile), all *p* < 0.05. Most improvement in clinical performance was due to reduced overtreatment of anemia, a decline in the percentage of patients with hemoglobin ≥ 12 g/dL; for-profits and facilities in African American neighborhoods had the greatest reduction.

**Conclusions:**

In the first year of CMS pay-for-performance, most clinical improvement was due to reduced overtreatment of anemia. Facilities in African American neighborhoods were more likely to receive a payment reduction, despite their large decline in anemia overtreatment.

## Background

In 2012, the United States Centers for Medicare and Medicaid Services (CMS) reported outcomes for its End-Stage Renal Disease Quality Incentive Program (ESRD QIP), a pay-for-performance (P4P) program for dialysis facilities. The ESRD QIP is instructive as a case-study of the ability of financial incentives to improve quality and impact disparities, and the challenges of creating policy in changing clinical and policy contexts.

The ESRD QIP builds on prior efforts to improve the quality and value of ESRD care. In 2001, CMS began publicly reporting dialysis performance measures on Dialysis Facility Compare, a public report card of facility performance. In 2008, US Congress passed the Medicare Improvements for Patients and Provider Act of 2008 (MIPPA) which established the ESRD QIP, a value-based purchasing or P4P program for Medicare that began in 2012 [1]. MIPPA also bundled payments to dialysis facilities starting in 2011 to reduce incentives to provide expensive erythropoietin stimulating agents (ESAs) due to high ESA costs and evidence of potential harms of ESAs at high doses [2–5].

The ESRD QIP reduces Medicare ESRD payments to dialysis facilities whose total performance scores do not meet or exceed national standards. Facilities with a total performance score less than 26 (out of a possible 30) have their Medicare payments for dialysis services reduced on a sliding scale, ranging from 0.5% to a maximum of 2%. Total performance scores and payments for 2012 are based on the dialysis facilities’ outcomes from 2010 for dialysis adequacy (percentage of patients with urea reduction ratio ≥ 65) and inadequate anemia management (both undertreatment, the percentage of patients on ESAs with hemoglobin (Hgb) < 10 g/dL, and overtreatment, the percentage of patients with Hgb > 12 g/dL).

Supporters of value-based purchasing or P4P programs contend that by measuring and reporting outcomes and linking them to reimbursement, dialysis facilities will have the data and motivation to improve their quality of care. Achieving targeted hemoglobin and dialysis adequacy is associated with decreased morbidity, mortality, and improved quality of life [6–10]. Similarly, randomized trials have shown that overtreatment of anemia (Hgb greater than 12 g/dL) in patients with chronic kidney disease is associated with increased mortality, partly due to increased erythropoietin [3]. In addition, because these outcomes are publicly reported online as part of Dialysis Facility Compare [11], consumers could make informed choices about dialysis facilities.

Critics counter that P4P might be ineffective in improving quality of care or, worse, have unintended negative consequences. P4P could be ineffective if the money at risk is not sufficient to encourage dialysis facilities to improve quality [12]. P4P could also exacerbate disparities in dialysis quality [13]. Dialysis facilities in neighborhoods with a higher proportion of African American residents have performed less favorably on quality indicators for anemia management and dialysis adequacy [14, 15]. P4P could worsen disparities if dialysis facilities “cherry-pick” healthier patients, leaving patients with more medical or social disadvantage in low-performing centers. P4P could also worsen racial or socioeconomic disparities if poorly performing dialysis facilities are chosen less by well-informed, well-insured patients, receive less reimbursement, and improve less than their already high performing counterparts.

Thus we examined if quality improved and disparities changed under QIP. First we explored what dialysis facility and neighborhood characteristics are associated with total performance scores that led to payment reductions under QIP. Then we investigated changes in QIP’s three clinical performance measures between 2007 and 2010.

## Methods

### Data

The Centers for Medicare and Medicaid Services (CMS) 2012 ESRD QIP Facility Performance contains information on all CMS certified dialysis facilities about anemia management and dialysis adequacy for 2010, metrics used for P4P in fiscal year 2012.To obtain demographic information for the neighborhood where the facility is located, as well as for state and region, we linked the CMS ESRD QIP file to the 2007–2011 American Community Survey (ACS) five-year summary file facility census tract by geocoding facility street address. We excluded facilities that did not have complete QIP score information (*n* = 483, 8.7%). For the addresses for which a street address could not be geocoded (*n* = 70, 1.3%), we used the census tract based on the zipcode XY centroid [16]. This study was deemed exempt by the IRB.

### Outcomes and co-Variates

#### Outcome variables

In the ESRD QIP, facilities were given a 1–10 rating for each of three criteria: the proportion of patients with Hgb less than 10 g/dL, the proportion of patients with Hgb greater than 12 g/dL (for the hemoglobin values, a lower proportion gives a better score), and the proportion of patients receiving adequate dialysis (urea reduction rate [URR] > 65%). Per QIP, the scores were summed so each facility received a score between 1 and 30 which was translated to a five-category measure of payment reduction for fiscal year 2012: (1) 0%, score 26–30; (2) 0.5%, score 21–25; (3) 1%, score 16–20; (4) 1.5%, score 11–15; and (5) 2%, score 10 or less.

We based our outcome measures on the ESRD QIP. We reverse coded adequate dialysis to measure inadequate dialysis, URR < 65, so that the change between 2007 and 2010 would move in a consistent direction. We collapsed 2% payment reduction into a category of 1.5% or more since there were few observations (*n* = 32). To determine which dialysis facility characteristics were associated with good and poor quality measures, we transformed categorical variables to two dichotomous outcome variables. Our first measure was ‘any payment reduction’ versus ‘no payment reduction,’ where any payment reduction represented the payment reduction percentage of 0.5% or more (coded as 1) and no payment reduction indicated 0% payment reduction (coded as 0). Our second dichotomous outcome was ‘most payment reduction’ versus ‘other payment reduction,’ where most payment reduction was defined as payment reduction percentage of 1.5% or greater (coded as 1) and other payment reduction was defined as 1% or smaller (coded as 0).

An additional outcome was change over time of the clinical outcome. To assess direct effects of QIP on quality improvement, we compared 2007 and 2010 performance for each dialysis facility on each of the three QIP outcomes.

#### Co-variates

We examined dialysis facility and neighborhood characteristics while accounting for ESRD network. Network describes the 18 ESRD Networks which are regional entities that contract with CMS and are responsible for organization, health planning, and quality improvement for ESRD care. Dialysis facility characteristics included profit status (i.e., for-profit versus non-profit), chain type (i.e., non-chain (independent), Chain 1, Chain 2, versus all other chains), facility size (i.e., the total number of dialysis stations at the dialysis facility by tertile), and years of operation (i.e., years since CMS certification). Chain membership is an organizational structure where a single firm owns a number of dialysis facilities, defined by USRDS as 20 or more freestanding facilities. Measures of dialysis facility neighborhood context were based on census tract. Census tract is the most commonly accepted proxy for neighborhood within social science and health services research [17]. Dialysis facility neighborhood-level characteristics included percentage of African American and percentage of population below Federal Poverty Level to represent neighborhood socioeconomic context [14, 15]. These variables were right-skewed; therefore, we divided them into quartiles to create categorical variables. All categorical variables (number of stations by tertile, percent African American by quartile, and percent poverty by quartile) were analyzed as indicator variables.

### Statistical analysis

Data were summed using descriptive statistics. We used a generalized linear mixed effects model to examine associations between each outcome and dialysis facility characteristics (facility type, length of operation, total number of stations) and facility neighborhood demographics (percent African American and percent of population below Federal Poverty Level by quartile). Each covariate of interest was evaluated separately in bivariable analyses; then they were examined simultaneously in multivariate analyses. We used logistic regression for binary outcomes (any payment reduction and no payment reduction); we used linear regression for paired data for change in clinical outcomes over time. All models included a random effect for ESRD network to account for clustering. We considered an interaction term between neighborhood demographics (proportion African American and proportion below poverty) for any payment reduction and large payment reduction because prior work showed different outcomes in African American and non-African American poor neighborhoods [15, 18]. All analyses were conducted using Stata, version 12.0 (Stata Corp, College Station, TX).

## Results

### Baseline characteristics

Of 5089 CMS certified dialysis facilities, the majority were for-profit (82.6%) and part of a chain (79.0%), Table 1. The average dialysis facility had 18 stations and had been operating for 15 years, although there was wide variation. On average, facilities were located in neighborhoods that were 18.2% African American, though this distribution was right skewed; mean proportion African American was less than 1% in Quartile 1 and 54% in Quartile 4. The facilities had a mean QIP score of 26.3, and 70.4% of facilities had 0% payment reduction.

**Table 1:** Baseline characteristics of hemodialysis facilities overall and by pay-for-performance payment group

Variable	Overall	Group 1 (0% reduction)	Group 2 (0.5% reduction)	Group 3 (1% reduction)	Group 4 (≥1.5% reduction)
N (% of total)	5089	3580 (70.3%)	811 (15.9%)	289 (5.7%)	409 (8.0%)
Facility level characteristics					
For-Profit (% For-Profit by Payment Group)^a^	4205	2991	672	235	307
	82.6%	83.6%	82.9%	81.3%	75.1%
Chain (% Chain by Payment Group)^a^	3676	2611	588	217	260
	79.0%	79.9%	79.0%	81.6%	68.8%
Chain 1	1321	968	220	60	73
	26.0%	27.1%	27.1%	20.8%	17.9%
Chain 2	1291	917	196	80	98
	25.4%	25.6%	24.2%	27.7%	24.0%
Independent	998 19.6%	682 19.1%	168 20.7%	50 17.3%	98 24.0%
Other Chain (Reference)	1477 29.0%	1011 28.3%	227 15.4%	99 6.7%	140 9.5%
Size (#stations)^a^	17.63	17.15	19.36	19.74	18.63
	[17.4,17.9]	[16.9,17.4]	[18.8,19.9]	[18.8,20.7]	[17.8,19.5]
Low (0–13)	1667 32.8%	1265 35.3%	203 25.0%	65 22.5%	134 32.8%
Medium (14–20)	1845 36.25%	1289 36.0%	304 37.5%	117 40.5%	135 33.0%
High (21–80)	1577 31.0%	1026 28.7%	304 37.5%	107 37.0%	140 34.2%
Length of Operation	14.65	13.74	16.82	17.35	16.43
years (95% CIs)^a^	[14.4, 14.9]	[13.4,14.02	[16.2,17.4]	[16.3,18.4]	[15.6,17.3]
Neighborhood level characteristics (mean, 95% CIs)					
Percent African American^a^	18.2%	17.0%	20.9%	22.5%	20.6%
	[17.5,18.8]	[16.3,17.8]	[19.1, 22.6]	[19.4, 25.5]	[18.0, 23.1]
Percent Poverty	18.5%	15.8%	16.7%	17.1%	17.5%
	[18.1,18.8]	[15.8,16.1]	[16.1,17.3]	[16.0,18.2]	[16.5,18.5]
Outcomes (mean, 95% CIs)					
Mean Total Performance Score*	26.3 [26.1, 26.4]	18.0 [17.6, 18.4]	19.3 [18.4, 20.2]	20.1 [18.5, 21.7]	19.2 [17.9, 20.6]

^a^ *p* < 0.05, difference across groups. *Pay-for-Performance Payment Categories: Total Performance Score (TPS) 26–30, 0% payment reduction; TPS 21–25, 0.5% payment reduction; TPS 16–20, 1% payment reduction; TPS less than 15, payment reduction ≥1.5%

### Bi-variable and multi-variable association between any payment reduction and facility and neighborhood covariates

In bivariable analysis compared to other chains (Table 2), two large chains were less likely to have any payment reduction (Chain 1, OR 0.78, 95% CI [0.66, 0.92]; Chain 2, OR 0.84, 95% CI [0.71, 1.0]). Conversely, dialysis facilities with any payment reduction were more likely to have had longer length of operation (OR 1.04 per year, 95% CI [1.03, 1.04]) and a medium or large number of stations (OR 1.41, 95% CI [1.21, 1.64] and OR 1.81, 95% CI [1.54, 2.12], compared to small). Similarly, facilities in neighborhoods with greatest proportion of poverty (OR 1.42, 95% CI [1.19, 1.70], highest quartile versus lowest quartile) and greatest proportion of African Americans (OR 1.52, 95% CI [1.26, 1.83], highest quartile versus lowest quartile) were also more likely to be in the payment reduction group.

**Table 2:** Bi-Variate and multi-variate association between any payment reduction and facility and neighborhood Co-Variates

Outcome: Any Payment Reduction vs. None	Bivariate	Multi-variate
Co-Variates	OR (95% CI)	OR (95% CI)
Facility level characteristics		
Chain Type		
Other Chain	Referent	Referent
Chain 1	0.78^a^ [0.66, 0.92]	0.77^a^ [0.65, 0.91]
Chain 2	0.84^a^ [0.71, 1.00]	0.74^a^ [0.62, 0.88]
Independent	1.01 [0.85, 1.21]	0.94 [0.78, 1.13]
Total # Stations (per station)		
Low (0–13)	Referent	Referent
Medium (14–20)	1.41^a^ [1.21, 1.65]	1.30^a^ [1.11, 1.53]
High (21–80)	1.81^a^ [1.54, 2.12]	1.42^a^ [1.20, 1.69]
Length of Operation (per year)	1.04^a^ [1.03, 1.04]	1.03^a^ [1.03, 1.04]
Neighborhood Level Characteristics		
Percent African-American		
Quartile 1 ≤ 8.6%	Referent	Referent
Quartile 2 8.6%–15.7%	1.07 [0.90, 1.28]	1.01 [0.84, 1.21]
Quartile 3 15.7% -25%	1.29^a^ [1.08, 1.55]	1.15 [0.96, 1.39]
Quartile 4 > 25%	1.52^a^ [1.26, 1.83]	1.25^a^ [1.02, 1.54]
Percent Below Federal Poverty Level		
Quartile 1 ≤ 1.7%	Referent	Referent
Quartile 2 1.7% - 7.2%	1.16 [0.98, 1.39]	1.08 [0.90, 1.29]
Quartile 3 7.2% - 24.9%	1.18^a^ [0.99, 1.41]	1.06 [0.88, 1.28]
Quartile 4 > 24.7%	1.42^a^ [1.19, 1.70]	1.15 [0.95, 1.39]

^a^ *p* < 0.05. Multivariable model includes profit status, number of stations by tertile, length of operation, and both percent African American and percent below Federal Poverty Level by facility neighborhood by quartile. Confidence intervals are at the 95% significance level

In multivariable analysis (Table 2), dialysis facilities with any payment reductions were more likely to have longer operation (OR 1.03 per year, 95% CI [1.03, 1.04]) and a medium or large number of stations (OR 1.31, 95% CI [1.11, 1.52] and OR 1.42, 95% CI [1.20, 1.69], compared to small). Similarly, facilities in neighborhoods with greater proportion of African Americans (highest versus lowest quartile, OR 1.25, 95% CI [1.02, 1.54]) were still more likely to be in the payment reduction group, even after controlling for percent poverty. The two large national chains, Chain 1 and Chain 2, were significantly less likely to have a payment reduction compared to other chains (OR 0.77, 95% CI [0.65–0.91] and OR 0.74, 95% CI [0.62, 0.88], respectively).

### Bi-variable and multi-variable association between greatest payment reduction and facility and neighborhood

Appendix Table 5 shows factors associated with being in the greatest payment reduction group ≥1.5%, the worst category. In bivariable analysis, facilities with longer length of operation (OR 1.02 per year, 95% CI [1.01, 1.03]) were more likely to be in the worst (≥1.5%) payment reduction group. Conversely, two national chains (Chain 1 OR 0.56, 95% CI [0.41, 0.75] and Chain 2, OR 0.73, 95% CI [0.55, 0.96]) had lower odds of being in the greatest payment reduction group.

In multivariable analysis, facilities with longer length of operation (OR 1.02 per year, 95% CI [1.00, 1.03]) were more likely to be in the greatest (≥1.5%) payment reduction group. Conversely, two large national chains, Chain 1 and Chain 2 (OR 0.56, 95% CI [0.41, 0.76] and OR 0.69, 95% CI [0.52, 0.91], respectively) had lower odds of being in the greatest payment reduction group compared to other chains. There was no significant interaction between neighborhood demographics (proportion African American and proportion below poverty) and between these variables and ESRD network (analysis not shown).

### Change in clinical outcomes between 2007 and 2010 and facility and neighborhood covariates

Table 3 shows mean change in clinical outcomes between 2007 and 2010 by facility and neighborhood. Across most categories, there was a small increase in percent of patients with Hgb below 10 g/dL, which indicates anemia undertreatment. There was a significant difference between change in Hgb < 10 between the no payment reduction group which showed a small reduction in proportion with Hgb < 10 and any payment reduction groups that show a successive increase in proportion with Hgb < 10. In addition, most facilities saw a small decrease in percent of patients with inadequate dialysis. The largest change in clinical outcomes from 2007 to 2010 is reduced anemia overtreatment shown by a decrease in proportion of patients with Hgb greater than 12 g/dL. Two large national chains had larger mean change compared to other chains or non-chains (independent) (−43.6 and −32.3 versus −30.9 and −27.4, respectively, *p* < 0.05).

**Table 3:** Mean change in clinical performance 2007–2010 by facility and neighborhood, and performance-level characteristics

Variable	Mean Change in Hgb <10 g/dL [95% CI]^b^	Mean Change in Hgb > 12 g/dL [95% CI]^b^	Change in URR < 65 [95% CI]^c^
N (%)	4259	4259	4191
All facilities	1.36 [1.25, 1.47]	−33.87 [−34.52, −33.22]	−0.80 [−0.97, −0.62]
Facility Level Characteristics			
Chain			
Others	1.57 [1.36, 1.78]	−30.94 [−32.25, −29.64]	−0.97 [−1.36, −0.57]
Chain 1	1.18 [1.00, 1.36]	−43.63^a^ [−44.75,-42.53]	−0.25^a^ [−0.54, 0.04]
Chain 2	1.25 [1.05, 1.45]	−32.32^a^ [−33.37,-32.27]	−0.96 [−1.24, −0.68]
Independent	1.42 [1.11, 1.73]	−27.39^a^ [−28.94,-25.84]	−1.03 [−1.51, −0.55]
Neighborhood Level Characteristics (mean, 95% CIs)			
Percent African-American			
Quartile 1 ≤ 8.6%	1.27 [1.03, 1.50]	−30.85 [−32.21, −29.49]	−0.32 [0.67, 0.04]
Quartile 2 8.6%–15.7%	1.32 [1.11, 1.53]	−33.01 [−34.26, −31.76]	−0.96 [−1.42, −0.51]
Quartile 3 15.7% -25%	1.47 [1.26, 1.69]	−34.97^a^ [−36.26,-33.68]	−0.80 [−1.12, −0.48]
Quartile 4 > 25%	1.36 [1.14, 1.58]	−36.48^a^ [−37.73,-35.22]	−1.06^a^ [−1.35, −0.76]
Percent Below Federal Poverty Level			
Quartile 1 ≤ 1.7%	1.31 [1.09, 1.53]	−33.19 [−34.50, −31.89]	−0.86 [−1.16, −0.55]
Quartile 2 1.7% - 7.2%	1.38 [1.16, 1.60]	−33.81 [−35.11, −32.52]	−0.79 [−1.16, −0.42]
Quartile 3 7.2% - 24.9%	1.30 [1.09, 1.50]	−34.52 [−35.83, −33.20]	−0.76 [−1.16, −0.37]
Quartile 4 > 24.7%	1.42 [1.18, 1.66]	−34.05 [−35.31, −32.79]	−0.76 [−1.12, −0.41]
Payment Reduction			
1 0%	−0.16 [−0.26, −0.06]	−34.50 [−35.38, −33.82]	−1.58 [−1.78, −1.37]
2 0.5%	2.27^a^ [2.09, 2.45]	−32.28 [−33.91, −30.65]	0.39^a^ [−0.04, 0.81]
3 1%	4.16^a^ [3.98, 4.35]	−33.13 [−35.80, −30.46]	1.11^a^ [0.51, 1.72]
4 ≥ 1.5%	7.78^a^ [7.44, 8.11]	−32.63 [−34.64, −30.62]	0.69^a^ [−0.04, 1.42]

^a^Significant Difference, *p* < 0.05 based on ANOVA. ^b^Hemoglobin (Hgb) goals: 10 ≤ Hgb ≤ 12, so decreasing proportion less than 10 or greater than 12 is a positive change. ^c^Adequate dialysis is Urea Reduction Rate (URR) greater than or equal to 65, so increasing proportion is positive change. Confidence intervals are at the 95% significance level

Table 4 shows multivariable results of facility and neighborhood characteristics associated with clinical outcomes over time. After adjusting for other factors, chain type (i.e., Chain 1) was associated with clinical improvement across all categories. Similarly, neighborhoods with greatest proportion of African Americans had significant improvement in two categories, decrease in percent of patients with Hgb > 12 g/dL and decreased percentage of patients with urea reduction rate less than 65. An analysis (not shown) examining random effects within our two-level model demonstrated larger variation our outcomes between facilities than between network. For example, for urea reduction rate less than 65 the network variance is 0.17 while the residual (facility) variance is 33.4. For Hgb > 12 g/dL, network variance is 0.03 and facility variance is 7.2; for Hgb < 10 g/dL network variance is 18.4 and facility variance is 408.1. The residual variance for facility was larger than for network across all three outcomes.

**Table 4:** Multivariable regression for change in clinical performance 2007–2010 by facility and neighborhood co-variates

Co-Variates	Change in Hgb <10 g/dL [95% CI]^b^	Change in Hgb > 12 g/dL [95% CI]^b^	Change in URR < 65 [95% CI]^c^
	Coefficients	Coefficients	Coefficients
Facility Level Characteristics			
Chain Type			
Other chain	Referent	Referent	Referent
Chain 1	−0.38^a^ [−0.68, −0.67]	−12.69^a^ [−14.41, −10.98]	0.91^a^ [0.42, 1.41]
Chain 2	−0.41^a^ [−0.72, −0.10]	−0.46 [−2.19, 1.26]	0.14 [−0.35, 0.64]
Independent	−0.08 [−0.41, 0.26]	3.72^a^ [1.84, 5.59]	−0.06 [−0.60, 0.49]
Total # Stations (per station)			
Low (0–13)	Referent	Referent	Referent
Medium (14–20)	−0.11 [−0.39, 0.17]	0.12 [−0.47, 1.70]	−0.33 [−0.79, 0.14]
High (21–80)	0.11 [−0.20, 0.42]	0.77 [−0.96, 2.49]	−0.37 [−0.87, 0.13]
Length of Operation (per year)	0.02^a^ [0.00, 0.03]	0.03 [−0.04, 0.11]	−0.003 [−0.03, 0.02]
Neighborhood Level Characteristics			
Percent African-American			
Quartile 1 ≤ 8.6%	Referent	Referent	Referent
Quartile 2 8.6%–15.7%	0.07 [−0.25, 0.39]	−1.22 [−3.00, 0.57]	−0.63^a^ [−1.15, −0.11]
Quartile 3 15.7% −25%	0.19 [−0.12, 0.55]	-2.42^a^ [−4.29, −0.54]	−0.53^a^ [−1.07, 0.01]
Quartile 4 > 24.7%	0.04 [−0.29, 0.45]	−3.43^a^ [−5.52, −1.34]	−0.85^a^ [−1.05, −0.27]
Percent Below Federal Poverty Level			
Quartile 1 ≤ 1.7%	Referent	Referent	Referent
Quartile 2 1.7% - 7.2%	0.06 [−0.25, 0.38]	−0.33 [−2.08, 1.43]	0.09 [−0.42, 0.60]
Quartile 3 7.2% - 24.9%	0.04 [−0.28, 0.37]	−0.53 [−2.35, 1.29]	0.19 [−0.34, 0.71]
Quartile 4 > 24.7%	0.13 [−0.21, 0.47]	0.80 [−1.10, 2.71]	0.30 [−0.25, 0.85]

^a^ *p* < 0.05. ^b^Hemoglobin (Hgb) goals: 10 ≤ Hgb ≤ 12, so decreasing proportion less than 10 or greater than 12 (negative sign) is a positive clinical change. ^c^Inadequate dialysis is Urea Reduction Rate (URR) <65 so decreasing proportion of URR < 65 (negative sign) is a positive clinical change. Confidence intervals are at the 95% significance level. Multivariable model includes profit status, total number of stations (by tertile), length of operation, percent African American by facility neighborhood (by quartile), percent below Federal Poverty Level by facility neighborhood (by quartile)

## Discussion

The first year of P4P for dialysis facilities has been a qualified success in the US. Several positive results are suggested by our data. The majority of dialysis facilities met CMS quality benchmarks related to dialysis adequacy and hemoglobin management and did not receive a payment reduction in 2012. However, total performance standards set for the 2012 payment year were modest at best. The measurement standards are based either on 2008 national benchmarks for each outcome or the actual 2007 results for each provider, whichever is lower. Thus, lower performing facilities were able to use their 2007 results for the baseline rather than being held to national performance standards.

Other clinical and regulatory forces were also at work. Announcements in 2008 about ESRD QIP and bundled payment, which reduced incentives to provide expensive erythropoietin stimulating agents (ESAs), occurred at the same time as evidence mounted of potential harms of ESAs at high doses [2, 3, 5]. High doses of ESAs to reach high hemoglobin levels were associated with greater risk for stroke, thromboembolic events, cardiovascular events, all-cause mortality and potentially cancer [2, 3, 5]. In 2007, the US Federal Drug Administration (FDA) issued a black-box warning for ESAs which called for using the lowest possible dose. As a result, many professional bodies changed their guidelines for ESA dosing for ESRD-related anemia [19–21]. Additionally, since 2001 CMS had been requiring dialysis facilities to monitor and report their outcomes. It is unclear whether the process of monitoring and reporting, or anticipating the potential financial penalties for QIP or bundled payment, led to improvement. Regardless of that uncertainty, a larger proportion of patients in dialysis facilities received guideline recommended care, which will likely lead to better clinical outcomes.

For-profit facilities were less likely to have received any payment reduction and were less likely to be in the largest payment reduction group. Those successes were largely due to two large national chains that represent more than half of US dialysis facilities. These large chains may have been better able to develop and disseminate effective clinical monitoring systems and clinical protocols. Our work found that the largest improvement was reduction in anemia overtreatment, and this change was significantly larger for Chain 1, a large for-profit national chain. Chain 1 also had small improvements in the two other categories (anemia undertreatment and dialysis adequacy). In prior work, for-profit facilities were associated with anemia overtreatment, higher than recommended hematocrit, and larger doses of erythropoietin-stimulating agents (ESAs), even after the FDA black box warning about ESAs’ harms at high doses [22, 23]. Adherence to clinical guidelines for ESAs, anticipation of bundled payments to facilities which now include ESAs, and QIP may have all contributed to this decline in anemia overtreatment [3, 5, 24, 25]. However, this result demonstrates how aligning payments—both bundled payments and ESRD QIP—with desired clinical outcomes can motivate capable organizations to change their practices.

When evaluating whether ESRD QIP increases disparities in quality, the evidence is also mixed. Facilities in predominately African American communities showed the greatest reduction in percent of patients overtreated for anemia and a more modest increase in percent of patients with adequate dialysis. Despite these improvements, in absolute terms, facilities in predominately African American neighborhoods still had lower total performance scores. ESRD QIP rewards absolute performance rather than relative improvement. Thus, the likelihood of a facility receiving any payment reduction increased as proportion of African Americans in the neighborhood increased, even after controlling for neighborhood poverty. Our work is consistent with the growing body of literature that recognizes neighborhood context (i.e., poverty or racial composition) as a contributor to disparities in quality of care for patients with ESRD [14, 18, 26]. The challenge of ESRD QIP, similar to other P4P programs, is how to incentivize quality improvement without widening quality or payment disparities [27, 28].

Efforts are underway to determine if and how to account for social factors that may lead to poor health care outcomes (e.g. low education, racial minority status, residence in disadvantaged neighborhood) in P4P programs without accepting lower quality for individuals in those groups [28]. Two distinct methods—risk adjustment and payment adjustment—attempt to measure and improve quality, while taking social factors into account to avoid unfairly penalizing particular providers or institutions. Risk adjustment determines what clinical and social factors should be accounted for when reporting outcomes; that is the quality benchmarks may be differ but payment for reaching one’s designated benchmark is the same. Payment adjustment sets the same quality benchmark for every institution, but adjusts payment based on accounting for clinical or social factors. The payments can be based risk adjusted, increased after accounting for social factors, or can directly fund programs to improve the quality of care for disadvantaged patients.

Our analyses have limitations. First, our study was observational. While our work shows that facility and neighborhood variation exists in quality of care, the etiology of this variation is still unclear. Further work is needed to examine how staff attitudes and practices, patient characteristics, and facility-level processes vary by high- and low-performers as well as by facility type, neighborhood, and region. In addition, our measures of dialysis facility neighborhood context were limited to census tracts. Dialysis facilities may be affected by patients and employees from a larger catchment area, and our outcomes may be more dependent on larger community infrastructure and resources [29]. In addition, patients in dialysis facilities may have greater disadvantage than the surrounding area as measured by percent African American and poverty [14]. Finally, we accounted for the effect of ESRD network overall, but we did not examine payment reductions by specific ESRD networks, states, or counties. While variation within these smaller areas may exist, we found that a greater proportion of variation in facility outcomes was explained by facility-level characteristics rather than region.

## Conclusions

Pay-for-performance has arrived for dialysis facilities in the US. Centers are likely reassured that this policy-change is not draconian; the majority of centers received no payment reduction. CMS policy makers are likely pleased at the improvement in anemia management. Most of the improvement appeared to be due to reduced anemia overtreatment without a large increase in anemia undertreatment. However, reports of the early successes of ESRD QIP must be tempered with caution. The large improvement in anemia management occurred with the concomitant incentives of P4P, anticipation of bundled payment for ESAs, and mounting clinical evidence of the potential harms of ESAs. In addition, facilities in largely African American communities still fared worse in this new era of P4P, despite significant quality improvement on dialysis adequacy and anemia overtreatment. In subsequent years, disparities may widen in the ESRD QIP as CMS increases the number of clinical outcomes measured and raises the thresholds that need to be met [30]. Outcomes must continue to be monitored to ensure that the program effectively improves quality of care for all patients with ESRD regardless of type of facility or neighborhood.

## Appendix

**Table 5:** Bi-Variate and multi-variate association between greatest payment reduction and facility and neighborhood Co-Variates

Outcome: Greatest Payment Reduction (≥1.5%) versus Other	Bi-Variate	Multi-variate
Co-Variates	OR	OR
Facility Level Characteristics		
Chain Type		
Other chain	Referent	Referent
Chain 1	0.56^a^ [0.41, 0.75]	0.56^a^ [0.41, 0.76]
Chain 2	0.73^a^ [0.55, 0.96]	0.69^a^ [0.52, 0.91]
Independent	1.16 [0.87, 1.54]	1.11 [0.83, 1.49]
Total # Stations (per station)		
Low (0–13)	Referent	Referent
Medium (14–20)	0.95 [0.73, 1.22]	0.94 [0.72, 0.22]
High (21–80)	1.21 [0.94, 1.56]	1.08 [0.82, 1.42]
Length of Operation (per year)	1.02^a^ [1.01, 1.03]	1.02^a^ [1.01, 1.03]
Neighborhood Level Characteristics		
Percent African-American		
Quartile 1 ≤ 8.6%	Referent	Referent
Quartile 2 8.6%–15.7%	0.98 [0.72, 1.32]	1.00 [0.74, 1.36]
Quartile 3 15.7% -25%	1.17 [0.87, 1.57]	1.17 [0.85, 1.59]
Quartile 4 > 25%	1.09 [0.80, 1.49]	1.06 [0.75, 1.50]
Percent Below Federal Poverty Level		
Quartile 1 ≤ 1.7%	Referent	Referent
Quartile 2 1.7% - 7.2%	1.29 [0.97, 1.72]	1.21 [0.91, 1.62]
Quartile 3 7.2% - 24.9%	0.98 [0.72, 1.33]	0.91 [0.66, 1.25]
Quartile 4 > 24.7%	1.24 [0.92, 1.66]	1.10 [0.79, 1.51]

^a^ *p* < 0.05 Confidence intervals are at the 95% significance level. Multivariable model includes profit status, total number of stations by tertile, length of operation, and both percent African American and percent below Federal Poverty Level by facility neighborhood (by quartile)

## Abbreviations

ACS: American community survey
CMS: Centers for medicare and medicaid services
ESA: Erythropoietin stimulating agent
ESRD: End stage renal disease
Hgb: Hemoglobin
MIPPA: Medicare improvements for patients and provider act of 2008
P4P: Pay-for-performance
QIP: Quality incentive plan
